# An Innovative Approach to Address Neurodegenerative Diseases through Kinase-Targeted Therapies: Potential for Designing Covalent Inhibitors

**DOI:** 10.3390/ph16091295

**Published:** 2023-09-13

**Authors:** Swapnil P. Bhujbal, Jung-Mi Hah

**Affiliations:** 1 College of Pharmacy, Hanyang University, Ansan 426-791, Republic of Korea; swapnil18@hanyang.ac.kr; 2 Institute of Pharmaceutical Science and Technology, Hanyang University, Ansan 426-791, Republic of Korea

**Keywords:** protein kinase, cancer, neurodegenerative diseases, covalent inhibitors

## Abstract

Owing to the dysregulation of protein kinase activity in various diseases such as cancer and autoimmune, cardiovascular, neurodegenerative, and inflammatory conditions, the protein kinase family has emerged as a crucial drug target in the 21st century. Notably, many kinases have been targeted to address cancer and neurodegenerative diseases using conventional ATP-mimicking kinase inhibitors. Likewise, irreversible covalent inhibitors have also been developed for different types of cancer. The application of covalent modification to target proteins has led to significant advancements in the treatment of cancer. However, while covalent drugs have significantly impacted medical treatment, their potential for neurodegenerative diseases remains largely unexplored. Neurodegenerative diseases present significant risks to brain function, leading to progressive deterioration in sensory, motor, and cognitive abilities. Alzheimer’s disease (AD), Huntington’s disease (HD), Parkinson’s disease (PD), and multiple sclerosis (MS) are among the various examples of such disorders. Numerous research groups have already reported insights through reviews and research articles on FDA-approved covalent inhibitors, revealing their mechanisms and the specific covalent warheads that preferentially interact with particular amino acid residues in intricate detail. Hence, in this review, we aim to provide a concise summary of these critical topics. This summary endeavors to guide medicinal chemists in their quest to design covalent inhibitors for protein kinases, specifically targeting neurodegenerative diseases.

## 1. Introduction

Kinases are a class of enzymes responsible for catalyzing the transfer of a phosphate group from a high-energy donor molecule (usually ATP) to a substrate molecule [1]. These enzymes are categorized based on the type of substrate they phosphorylate, leading to two main groups: metabolic kinases and protein kinases. The protein kinases are further divided into three subcategories: tyrosine-specific kinases, serine/threonine-specific kinases, and mixed-specificity kinases [1,2,3]. They play a crucial role in regulating the intracellular signaling pathways that govern vital cellular processes, such as cell proliferation, survival, differentiation, and apoptosis. Both protein and metabolic kinases hold vital significance in numerous human diseases, encompassing immune disorders and cancers [3]. The dysregulation of kinase activity has been linked to over 400 diseases [4]. For example, mutations in genes encoding protein kinases can lead to excessive kinase activity. To address this, several clinically approved drugs, such as selective kinase inhibitors, have been designed to target and inhibit the enzymatic activity of specific protein kinases [1,2,3,4,5]. The FDA-approved kinase inhibitors encompass conventional acyclic, macrocyclic, and covalent inhibitors, all developed to enhance efficacy and selectivity against their respective kinase targets. This review specifically concentrates on exploring opportunities for designing covalent kinase inhibitors to treat neurodegenerative diseases.

### 1.1. Protein Kinases in Drug Discovery

Due to overexpression and genetic alterations, such as mutations and translocations, protein kinase activity becomes dysregulated. This contributes to the pathogenesis of numerous diseases, including autoimmune, cardiovascular, nervous, and inflammatory conditions, as well as various malignancies. Consequently, the kinase family has emerged as a crucial drug target in the 21st century [6,7], with approximately one-quarter of the world’s drug discovery efforts focusing on protein kinases [8]. Remarkably, there are currently 62 FDA-approved therapeutic agents that target around two dozen distinct protein kinases [8]. The public domain hosts over five thousand protein kinase structures, serving as valuable resources for structure-based drug development. Additionally, the pharmaceutical industry holds a considerable number of proprietary structures utilized in the drug development process. The ongoing clinical trials worldwide involve about 175 different orally effective protein kinase antagonists [9], and a comprehensive listing of these drugs, regularly updated, can be accessed at www.icoa.fr/pkidb/ (accessed on 16 June 2023). Despite these impressive numbers, it is worth noting that the 62 FDA-approved therapeutic agents and the additional drugs in clinical trials only target a fraction of the vast 518-member protein kinase enzyme family [5,9].

#### 1.1.1. Protein Kinases in Neurodegenerative Diseases

Neuroregeneration refers to the processes of regeneration within the nervous system, encompassing the generation of new neurons, axons, glia, and synapses [6,7,10]. It involves the progressive structural and functional recovery of the damaged nervous system over time. Damage to the central nervous system (CNS) can result from cell death, failure of axonal regeneration, demyelination, and overall deficits in neuronal structure and function [5]. These conditions, whether partial or complete, occurring individually or in combination, and whether arising from genetic factors or acquired, collectively give rise to specific neurological disorders known as neurodegenerative disorders. These neurodegenerative disorders pose significant threats to the normal functioning of the brain, leading to gradual deterioration or even total loss of sensory, motor, and cognitive function. Examples of such disorders include Alzheimer’s disease (AD), Huntington’s disease (HD), Parkinson’s disease (PD), and multiple sclerosis (MS), among others [5].

Significantly, neurodegenerative diseases are characterized by the abnormal accumulation of proteins in the brain or tissue. For instance, Alzheimer’s disease (AD) involves the buildup of β-amyloid, while Huntington’s disease (HD) is associated with misfolded Huntington protein aggregation. In amyotrophic lateral sclerosis, there is an aggregation of ubiquitinated proteins [11]; multiple sclerosis (MS) plaques exhibit the accumulation of Tau and β-amyloid [8]; while Parkinson’s disease (PD) is marked by α-synuclein accumulation. Additionally, traumatic brain injuries are linked to Tau neurofibrillary tangles [12]. Compelling evidence indicates that the spread of misfolded proteins from one cell to another contributes to the disease’s progression [5,13]. Currently, there are no cures for neurodegenerative diseases, especially in their advanced stages. However, the development of therapeutic approaches remains of paramount importance to address physiological and cognitive deficits [5].

Neurodegenerative diseases impact distinct regions of the brain, exhibiting unique and evident characteristics at the phenotypic level, such as the progressive loss of sensory–motor and cognitive functions [14]. However, despite these differences, they share similar cellular and molecular etiologies [15]. Analyzing these commonalities offers the potential for therapeutic advancements that could simultaneously address multiple neurodegenerative disorders if we gain a clear understanding of their shared features [5,16]. Among these, Alzheimer’s (AD), Parkinson’s (PD), Huntington’s disease (HD), and multiple sclerosis (MS) are the most prevalent forms. AD stands as the leading cause of dementia worldwide, severely impacting an individual’s ability to perform everyday activities. In the United States alone, an estimated 5.4 million people are affected by AD, including approximately 200,000 individuals aged under 65, making up the younger-onset AD population [17]. The harmful etiological factors in AD involve the buildup of β-amyloid protein and intracellular aggregation of tau protein, which may trigger synaptopathies, glial inflammation, and eventual neuronal death in the cerebral cortex, sub-cortical regions, temporal and parietal lobes, and cingulate gyrus [5].

Both AD and PD are progressive diseases associated with the intracellular accumulation of toxic protein aggregates. Despite their distinct and evident phenotypic characteristics, they share a common etiology at the subcellular level [18,19]. However, the current treatment approaches for neurodegenerative diseases only target a small subset of the population and primarily focus on providing symptomatic relief, lacking the ability to alter the progression of the diseases [5,19]. Using a covalent kinase inhibitors (CKI) strategy in the design of new kinase inhibitors presents a compelling and innovative solution to address these challenges. CKI have been developed and reported as promising drug candidates for various diseases, with a particular emphasis on their therapeutic applications in the field of oncology [18,19]. Therefore, it is advisable to develop novel CKI-targeting kinases for which only anticancer inhibitors had been previously documented, for example, JNK3 (c-Jun N-terminal kinase 3).

#### 1.1.2. JNK3 in Neurodegenerative Diseases

JNKs, part of the mitogen-activated protein kinases (MAPKs) family, are stress-activated serine-threonine protein kinases [20]. They play a crucial role in regulating various cellular activities, ranging from proliferation to cell death [21]. While JNK1 and JNK2 are widely expressed in all body tissues, JNK3 expression is restricted to the central nervous system (CNS), cardiovascular system, smooth muscle, and testis [21]. JNK3 primarily participates in neurodegenerative processes, including Alzheimer’s disease (AD), Parkinson’s disease (PD), cerebral ischemia, and other CNS disorders. Significantly, JNK3 has been detected in the cerebrospinal fluid (CSF) of AD patients, and its elevated levels have been statistically correlated with the rate of cognitive decline. This finding emphasizes JNK3’s crucial role as a key player in AD, making it an attractive target for CNS drug development [20,21]. Moreover, JNK3 plays a pivotal role in the initial neurodegenerative event, known as synaptic dysfunction, which leads to the perturbation of physiological synapse structure and function. Excitingly, synaptic dysfunction and spine loss have been shown to be pharmacologically reversible, offering promising therapeutic directions for brain diseases. Additionally, JNK3 could serve as a valuable biomarker for disease detection, as its presence can be detected at the peripheral level, enabling early diagnosis of neurodegenerative diseases in their prodromal stages [20,21,22].

**Figure 1:**
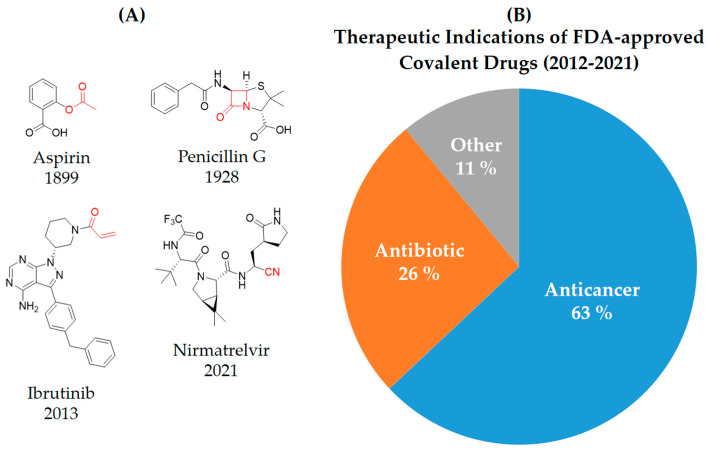
(**A**) Chemical structures of representative covalent inhibitors in early and recent years. (**B**) Therapeutic indications of covalent inhibitors [18].

Given this information, it is worth considering JNK3 as an illustrative example since it has not been extensively studied as a target for the development of covalent inhibitors in the past. Targeting protein kinases like JNK3 could prove advantageous in the design and advancement of covalent inhibitors for the treatment of neurodegenerative disorders. With the wealth of existing 3D structures of JNK3 and comprehensive information on covalent inhibitors and their warheads, the enticing advantages and disadvantages have motivated us to embrace the challenge of designing covalent inhibitors for JNK3. Our aim is to target diseases like Alzheimer’s while ensuring these inhibitors possess blood–brain barrier (BBB) permeability. Furthermore, aside from its involvement in neurodegenerative diseases (ND), JNK3’s binding site contains a critical residue, cysteine154, which can form covalent bonds with most of the covalent warheads (as shown in Figure 1). This characteristic makes JNK3 an attractive target for the development of covalent inhibitors.

### 1.2. Recent Studies on Neurodegenerative Diseases

Limited research endeavors have been commenced in the realm of neurodegenerative diseases, particularly those targeting diverse macromolecules. Instances of investigations focusing on kinases as targets are relatively scarce. Some of these studies are briefly outlined hereafter. Yun et al.’s study revealed the significant involvement of the NLRP3 inflammasome in innate immune-mediated inflammation, contributing to the development of various neurodegenerative diseases. Despite this, there is currently a lack of clinically available medications targeting the NLRP3 inflammasome. Although RRx-001, an anticancer agent under phase III clinical investigation, is known for its well-tolerated nature, its potential impact on inflammatory conditions remains unexplored. In light of this, Yun et al. demonstrated the remarkable potential of RRx-001 as a specific and potent NLRP3 inhibitor, yielding substantial benefits for NLRP3-driven inflammatory ailments. Mechanistically, RRx-001 forms a covalent bond with Cysteine409 of NLRP3 through its bromoacetyl group, effectively obstructing the crucial NLRP3-NEK7 interaction necessary for NLRP3 inflammasome assembly and activation [23].

Bratkowski et al. identified a significant revelation regarding the role of nicotinamide adenine dinucleotide (NAD) hydrolase SARM1, positioning it as a pivotal metabolic sensor and executor of axonal functions. This finding presents a captivating avenue for the creation of innovative neuroprotective treatments aimed at impeding or arresting the degenerative progression. They elucidated a category of SARM1 inhibitors reliant on NAD that act within the active site, effectively intercepting NAD hydrolysis and engaging in covalent bonding with the resulting product, adenosine diphosphate ribose (ADPR) [24]. Moreover, Huang et al. have documented that the phosphoinositide 3-kinase (PI3K)/protein kinase B (AKT)/mammalian target of rapamycin (mTOR) pathway holds significant importance as a therapeutic target for neurodegenerative disorders. Their examination encompassed recent advancements in ATP-competitive inhibitors, allosteric inhibitors, covalent inhibitors, and proteolysis-targeting chimeras within the context of the PI3K/AKT/mTOR pathway. They highlighted potential strategies to counteract the toxicities and acquired drug resistance associated with current treatments and put forth recommendations for the future formulation and development of promising agents targeting this pathway [25]. We have not discussed these studies extensively in our review because comprehensive outcomes and elucidations for these investigations are available within their respective referenced articles, mentioned earlier.

## 2. Covalent Inhibitors

Covalent inhibitors are extensively used as both biochemical tools and therapeutic agents, allowing for the targeting of proteins previously considered “undruggable” [18,26]. The advanced selectivity of modern covalent inhibitors has significantly alleviated concerns about potential toxicity arising from protein covalent modifications. Despite the remarkable clinical success achieved by current covalent inhibitors, there still remain unmet medical needs that require further attention. The application of covalent drugs has been instrumental in improving human lives by utilizing the covalent modification of target proteins [18,26]. Iconic examples of covalent drugs include aspirin, a well-known anti-inflammatory medication, and penicillin, one of the earliest antibiotics to be developed (Figure 1A) [27]. Historically, the discovery of covalent drugs has been largely serendipitous, providing only a limited window of opportunity for exploring the landscape of covalent inhibitors. However, in recent decades, advancements in crystallography, computer-aided drug design, and modern organic chemistry have made the rational design of covalent inhibitors more feasible and efficient [18,26].

The conventional mechanism of covalent inhibitors, from aspirin to nirmatrelvir (Figure 1A), has involved targeting the catalytic residues of pathological enzymes. However, when these traditional covalent inhibitors encounter highly conserved residues across protein family members, they may lack subtype selectivity. In 2011, Singh and colleagues introduced a novel strategy utilizing targeted covalent inhibitors (TCIs) [28]. This approach achieves subtype selectivity by focusing on poorly conserved and non-catalytic residues. A notable example of TCIs is Ibrutinib (Figure 1A), a first-in-class BTK inhibitor approved for the treatment of mantle cell lymphoma and chronic lymphocytic leukemia (CLL). The clinical success of Ibrutinib has propelled the utilization of TCIs as the preferred approach in the development of covalent drugs [18]. The majority of FDA-approved TCIs exhibit selective inhibition of kinases by targeting poorly conserved cysteine residues near ATP-binding pockets. However, it is essential to note that such targetable cysteine residues are not widespread in the human proteome [29]. Due to this limitation, lysine has gained attention as a promising target residue for covalent modification due to its prevalence in the human kinome [18].

Despite their promising therapeutic potential, covalent drugs currently constitute only 4.4% of the drugs approved by the U.S. Food and Drug Administration (FDA) in the past decade [30]. Among these covalent drugs, 90% function as anticancer or antibiotic agents (Figure 1B). FDA-approved covalent kinase inhibitors are listed in Table 1.

## 3. Types of Covalent Inhibitors

**Figure 2:**
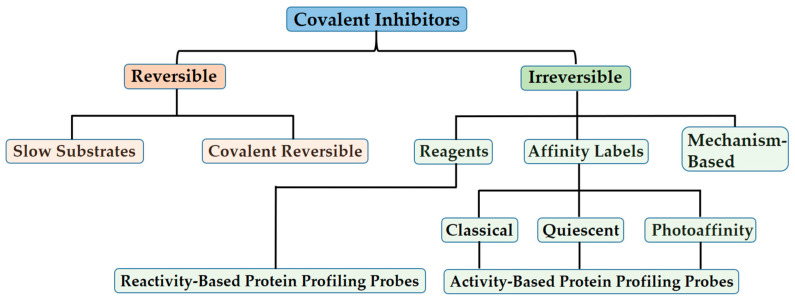
Taxonomy of covalent inhibitors. The classification of covalent enzyme inhibitors according to their mechanisms highlights diverse approaches aimed to balance reactivity and selectivity [26].

In addition to categorizing covalent inhibitors based on reactive groups or modification sites, Alfred Tuley et al. [26] and Jesang Lee et al. [18] introduced a classification based on a mechanism depicted in Figure 2. This method emphasizes the common advantages and disadvantages inherent to each approach. The established categories for reversible and irreversible covalent inhibitors are briefly outlined below, along with representative examples for each class, including covalent reversible inhibitors, slow substrates, residue-specific reagents, affinity labels (classical, quiescent, and photoaffinity), and mechanism-based inactivators. For a more comprehensive understanding of these types of covalent inhibitors, interested readers are encouraged to refer to the detailed works by Lee et al. [18] and Tuley et al. [26].

In general, covalent inhibitors can be broadly classified into two categories based on whether the inhibition is reversible or irreversible upon dialysis, competition with excess substrate, or extended incubation times. While different covalent inhibitors may exhibit the same reversibility or irreversibility of inhibition, each employs distinct strategies to selectively form and break bonds with the targeted enzyme. Identifying and categorizing these strategies helps to illuminate the inherent advantages and disadvantages associated with each mechanism [18,19,20,21,22,26].

### 3.1. Covalent Reversible Inhibitors

This class of inhibitors employs covalent bond formation with the enzyme to achieve inhibition, but enzyme activity is subsequently restored through the cleavage of this bond [2,3]. Covalent bond formation contributes to the overall affinity of the inhibitor, but the subsequent bond cleavage can decrease the duration of inhibition and lower the affinity [4]. This approach helps to mitigate some of the potential drawbacks associated with the formation of irreversible covalent adducts, but, at the same time, makes it more challenging to identify the proteins bound by these inhibitors. The most prevalent type of covalent inhibitor exhibiting reversible inhibition is named after this category because the term “reversible” accurately describes the underlying chemical mechanism [18].

### 3.2. Covalent Irreversible Inhibitors

The second major category of covalent inhibitors comprises those that induce irreversible inhibition (Figure 2). These inhibitors are also referred to as inactivators to underscore the irretrievable loss in enzyme activity they cause [26]. Since covalent modification by these inhibitors is not reversible, they cannot rely on thermodynamic equilibration to achieve selectivity. Instead, they employ different strategies to achieve selective modification. While forming an irreversible covalent adduct comes with some inherent disadvantages, this type of inhibitor offers the advantage of long-lasting inhibition that persists even as the inhibitor concentration in solution decreases. Additionally, irreversible inhibitors facilitate both target and off-target identification. Over time, the slow accumulation of inhibited enzymes, even in the presence of competing ligands, further contributes to the effectiveness of irreversible inhibitors [18,26].

In summary, it is important to highlight that, when dealing with this class of inhibitors, the use of IC_50_ values alone to guide structure–activity studies can be misleading due to the time-dependent nature of inhibition [31,32,33]. To accurately assess the inhibitory effects, parameters such as *K_I_*, which describes the noncovalent binding affinity (denoted with a capital I to differentiate it from *K_i_* values for reversible noncovalent inhibitors), and *k_inact_*, which describes the kinetics of covalent modification, can be utilized. These parameters can be derived from fitting time-dependent inhibition studies through various methods described elsewhere [34,35]. Irreversible inhibitors are divided into three main categories: residue-specific reagents, affinity labels, and mechanism-based enzyme inactivators.

#### 3.2.1. Residue-Specific Reagents

These are the least selective type of irreversible inhibitor, and they are primarily employed in vitro as biochemical tools [26]. These reactive compounds achieve selective modification by depending on chemoselectivity for specific nucleophiles, rather than noncovalent affinity to a particular binding site. The selectivity is also influenced by the relative nucleophilicity and steric availability of the reacting residues in the targeted proteins. Recently, many reagents used for residue-specific modification in proteins and peptides have been extensively reviewed [36]. Due to their limited noncovalent affinity for a specific protein site, these reagents typically exhibit second-order inactivation kinetics. A pertinent illustration of a residue-specific chemical modifying agent employed as a covalent inhibitor involves the use of methylmethanethiosulfonate to interact with cysteine residues in soluble guanylate cyclase. This process leads to the covalent attachment of methanethiol moieties through disulfide bonds to multiple cysteine residues located throughout the protein [37].

#### 3.2.2. Affinity Labels

Rather than relying solely on chemoselectivity, affinity labels increase their site selectivity by coupling a reactive group, typically a poor electrophile, to a second moiety that provides noncovalent binding affinity to a specific binding site [38]. Each of the three different types of affinity labels within this class increases the effective molarity of the reactive group near the site of enzyme modification by using a moiety to provide noncovalent binding. However, the strategy used to attenuate reactivity varies by type and can impact selectivity [26]. In contrast to relying solely on chemoselectivity, affinity labels enhance their site selectivity by combining a reactive group, usually a poor electrophile, with a second moiety that provides noncovalent binding affinity to a specific binding site [38]. The three different types of affinity labels (classical, quiescent, and photoaffinity) within this class employ a moiety to provide noncovalent binding, thus increasing the effective molarity of the reactive group near the site of enzyme modification. However, the approach used to attenuate reactivity differs for each type, and this variation can influence the overall selectivity.

#### 3.2.3. Covalent Mechanism-Based Enzyme Inactivators

Among all covalent enzyme inhibitors, those falling into the category of mechanism-based enzyme inactivators have the potential to be the most selective because they initially exist as unreactive molecules. These inhibitors bind to the active sites of enzymes and undergo normal catalytic processes, leading to the formation of a reactive species that results in covalent bond formation. The parameters *K_I_* and *k_inact_* represent a combination of individual rate constants, which, in certain cases (e.g., rapid equilibrium, *k*_4_ = 0, *k*_2_ is rate-limiting), can be simplified to represent the dissociation constant for the noncovalent complex and *k*_2_, respectively [39].

## 4. Advantages and Disadvantages of Covalent Inhibitors

Basically, a covalent bond is formed when atoms equally share electron pairs. In the context of covalent kinase inhibitor (CKI) development, this usually involves a ligand electrophile (e.g., acrylamide) and a target nucleophile (e.g., a cysteine sulfhydryl). However, achieving selective kinase targeting poses challenges, and there is a risk of cytotoxicity due to extended or permanent off-target modifications [40]. In short, the following are some of the potential advantages associated with covalent inhibitors.

### 4.1. Potency

Within a covalent inhibitor, the covalent attachment plays a crucial role in contributing to the overall free energy of binding (GB), leading to significantly enhanced potency while maintaining a low molecular weight. Unlike non-covalent inhibitors, covalent therapeutics are less affected by competition with high concentrations of endogenous substrates (e.g., ATP). This advantage is due to their reliance on non-equilibrium binding kinetics [41].

### 4.2. Pharmacodynamics (PD)

When the target is appropriately chosen, covalent inhibitors can exhibit a prolonged duration of action compared to their non-covalent counterparts. This leads to a separation between pharmacodynamics (PD) and pharmacokinetics (PK), referred to as PK-PD decoupling. As a result, covalent inhibitors can maintain sustained efficacy beyond metabolic clearance [40].

### 4.3. Drug Dosing

Covalent binders offer a clinically relevant advantage due to their high potency, sustained action, and decoupled PK-PD effect. This advantage allows for the administration of smaller and less frequent doses as compared to non-covalent drugs. These relatively smaller doses often result in a significant reduction in negative side effects, particularly those arising from unpredictable idiosyncratic toxicities (IDTs). In contrast, many non-covalent drugs may require more frequent and larger doses to maintain therapeutically efficacious plasma concentrations [40,42].

### 4.4. Drug Resistance

The primary source of resistance typically arises from mutations within the ATP binding site. Recent studies on covalent inhibitors have confirmed their capacity to hinder and evade mutation events, enabling them to maintain potency against mutant targets [40,43]. These investigations have suggested that while active-site mutations may impede the initial reversible binding (i.e., K_i_), subsequent covalent bond formation (i.e., k_inact_) can still occur, assuming that the reactive residue remains unaltered. However, it is essential to consider the vulnerability of covalent kinase inhibitors (CKIs) to mutations of the nucleophilic residue. In such cases, the potency can be significantly reduced due to the critical role of covalent bond formation in the observed efficacy.

### 4.5. Target Scope

CKIs heavily depend on covalent interactions for their potency. In specific cases, due to their reduced reliance on non-covalent intermolecular interactions, covalent drugs may offer the advantage of targeting challenging active sites, such as those that are poorly defined, large, and solvent-exposed. The nature of a covalent inhibitor allows for high potency without requiring extensive binding surface contact, which can lead to a smaller-sized molecule. Moreover, target selectivity is a critical consideration in any drug discovery program, and covalent compounds can be deliberately designed to bind to poorly conserved target-specific nucleophilic residues [40,44].

## 5. Disadvantages

Despite their advantages, covalent inhibitors also come with potential drawbacks that need to be considered. These include the increased challenge of evaluating and achieving a balance between reactivity and selectivity. Covalent modification may extend to off-target proteins, nucleic acids, or small molecules due to nonselective reactions. Moreover, idiosyncratic adverse drug responses, inappropriate levels of enzyme inhibition when partial or short-duration inhibition is required, and limitations in achieving some of the benefits mentioned earlier are also among the concerns. Additionally, designing these inhibitors de novo may be perceived as challenging [26,40].

## 6. Need for Covalent Kinase Inhibitors

### 6.1. Importance of Cysteine Residue

Covalent compounds that react with cysteine have seen a resurgence as potent and selective tools for modifying protein function, serving both as chemical probes and clinically approved drugs [2]. The remarkable sensitivity of human immune cell signaling pathways to oxidative stress suggests that covalent probes hold significant potential for selective chemical immunomodulation, an area that is still relatively underexplored [45,46]. Recently, cysteine-reactive compounds have regained prominence as powerful tools for altering protein function, especially for challenging-to-target protein classes [2]. These compounds, often referred to as covalent compounds, contain electrophilic moieties that react either irreversibly or reversibly with the thiol side chain of specific cysteine residues. The preferential labeling of specific cysteine results from a combination of factors, including the intrinsic reactivity of the thiol, the nature and relative reactivity of the electrophile, and the molecular recognition of the binding portion of the molecule [47].

**Figure 3:**
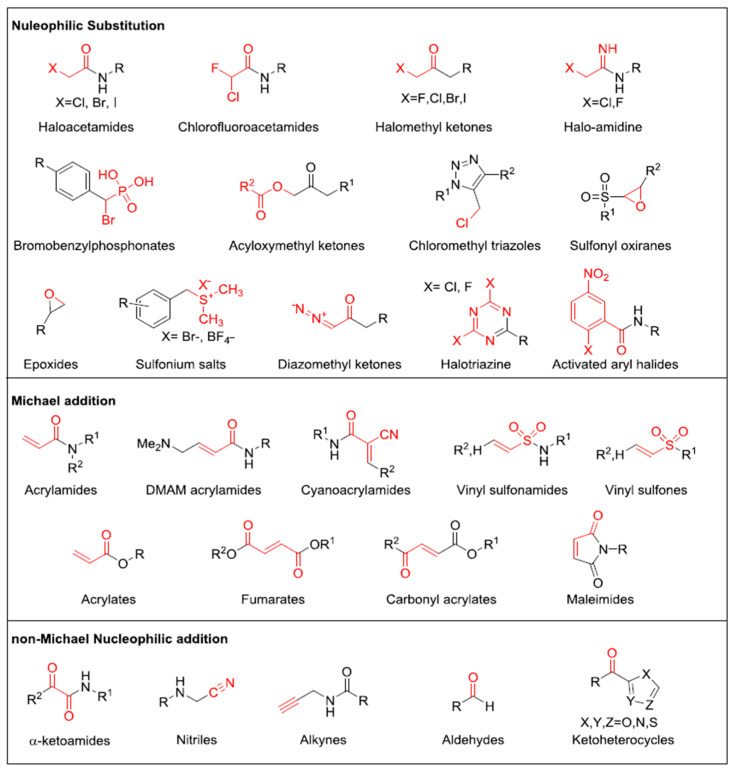
Commonly used cysteine-reactive electrophiles. Electrophilic groups, depicted in red, are categorized based on their specific mechanisms of covalent cysteine labeling.

Cysteine presents an intriguing amino acid target for several compelling reasons. The unique chemistry of the cysteine thiol renders cysteine residues crucial for the structure and function of most human proteins [2]. Cysteines often serve as catalytic nucleophiles in enzymes, such as proteases. Additionally, cysteines are involved in coordinating metals, forming structural and redox-active disulfides, undergoing frequent post-translational modifications, and acting as sensors of oxidative stress [2]. Cysteine-reactive compounds can be skillfully designed to access small and less-defined binding sites, allowing them to efficiently block high-affinity interactions, such as protein–protein interactions, or compete with high concentrations of endogenous biomolecules like ATP [47]. There exists a wide array of examples of cysteine-reactive clinical candidates and drugs, including blockbuster covalent kinase inhibitors (CKIs); anti-cancer compounds like KPT3 [48], which reacts with a conserved cysteine in the nuclear export factor XPO1 [49]; and ARS-1620, which inhibits the Gly12Cys-mutated oncogenic form of the GTPase KRAS [50]. Remarkably, nearly all human proteins contain at least one cysteine (with an average of 13 cysteines per protein), and recent studies [2,47] indicate that a surprisingly significant portion of cysteines can react with cysteine-reactive compounds [2,47]. Commonly used cysteine-reactive electrophiles are depicted in Figure 3, below. In our review, we do not provide a detailed description of how each of the cysteine-reactive electrophiles reacts, as this information was already covered in a previously published article [2,47].

### 6.2. Opportunities for Covalent Kinase Inhibitors (CKIs) by Targeting Kinases

Developing selective inhibitors for kinases poses a considerable challenge due to their high sequence and structural homology. Surprisingly, more than 200 members of the human protein kinase family have been found to harbor active site cysteines [51]. Targeting these residues with covalent kinase inhibitors (CKIs) offers an exciting strategy to create highly potent and selective ATP competitive kinase inhibitors, a topic covered extensively in recent comprehensive reviews [40]. CKIs have gained significant attention for their therapeutic efficacy in treating various cancers [2,40]. While many of the targeted kinases have clear immune-relevance, the full immunotherapeutic potential of CKIs is yet to be fully explored. To date, all identified CKIs primarily target protein kinases for cancer treatment; however, in this review, we focused on the opportunity to target kinases for neurodegenerative diseases. As some kinases exhibit higher degrees of promiscuous binding, an alternative strategy is to produce compounds targeting the inactive forms of kinases. However, complications arise, as some kinases can bind inhibitors more promiscuously when in their inactive conformation, making it challenging to generate selective inhibitors for inactive kinases, at least in certain cases.

Given this information, it is worth considering JNK3 as an illustrative example since it has not been extensively studied as a target for the development of covalent inhibitors in the past. Moreover, aside from its involvement in neurodegenerative diseases (ND), JNK3’s binding site contains a critical residue, cysteine154, which can form covalent bonds with most of the covalent warheads. Targeting protein kinases like JNK3 could prove advantageous in the design and advancement of covalent inhibitors for the treatment of neurodegenerative disorders. Presently, our research group is actively engaged in designing and synthesizing covalent inhibitors specific to JNK3. However, we cannot disclose the chemical structures of these designed covalent inhibitors in this review. Instead, we intend to publish them as a separate research article in the near future.

## 7. Conclusions

Due to the dysregulation of protein kinase activity in various diseases, including cancer, autoimmune, neurodegenerative, and inflammatory conditions, the protein kinase family has emerged as a crucial drug target. In this review, we have extensively discussed previously approved covalent inhibitors, elucidating their mechanisms and the specific covalent warheads that typically react with particular amino acid residues, most of which were developed for cancer treatment. However, the potential of these inhibitors for neurodegenerative diseases remains largely unexplored. We have also highlighted the advantages and disadvantages of covalent kinase inhibitors (CKIs) over conventional kinase inhibitors. Moreover, our focus is on the importance of designing CKIs for specific protein kinases, such as JNK3, which plays a significant role in neurodegenerative diseases like Alzheimer’s. We aim to help in the development of covalent inhibitors with high selectivity and improved blood–brain barrier (BBB) permeability. The concise summary provided in this review is intended to serve as a guide for the research community in their efforts to design covalent inhibitors for protein kinases, specifically targeting neurodegenerative diseases.

## Figures and Tables

**Table 1:** FDA approved covalent kinase inhibitors.

Name/Structure	Targets	Therapeutic Indication	Warhead	Ref (Approval Date)
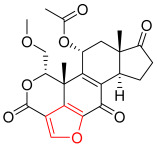 Wortmannin	Kinase (PI3K)	N/A	Furan	[24]
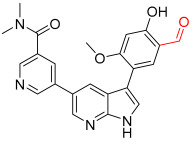 A5	Kinase (BCR-ABL)	Anticancer (chronic myeloid leukemia)	Aldehyde	[28]
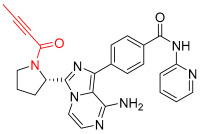 Acalabrutinib	Kinase (BTK)	Anticancer (mantle cell lymphoma	2-butyneamide	31 October 2017
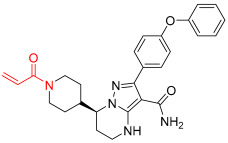 Zanubrutinib	Kinase (BTK)	Anticancer (mantle cell lymphoma	Acrylamide	14 November 2019
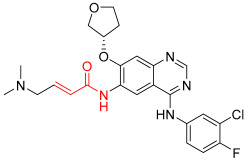 Afatinib	Kinase (EGFR T790M and pan-HER)	Anticancer (NSCLC)	Acrylamide	12 July 2013
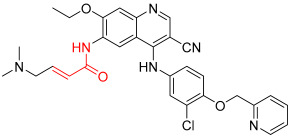 Neratinib	Kinase (pan-HER)	Anticancer (breast cancer)	Acrylamide	17 July 2017
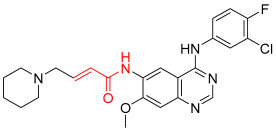 Dacomitinib	Kinase (pan-HER)	Anticancer (NSCLC)	Acrylamide	27 September 2018
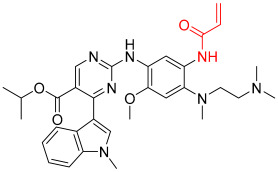 Mobocertinib	Kinase (EGFR ex20ins)	Anticancer (NSCLC)	Acrylamide	15 September 2021
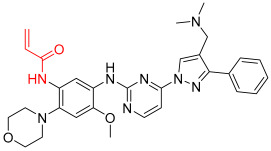 Lazertinib	Kinase (EGFR)	Anticancer (NSCLC)	Acrylamide	Accelerated approval, 21 May 2021, combination with amivantamab
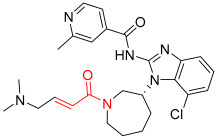 Nazartinib	Kinase (EGFR)	Anticancer (NSCLC)	Acrylamide	[29]

## Data Availability

Data sharing is not applicable.
